# Maternal tadalafil therapy for fetal growth restriction prevents non-alcoholic fatty liver disease and adipocyte hypertrophy in the offspring

**DOI:** 10.1038/s41598-020-80643-0

**Published:** 2021-01-13

**Authors:** Takuya Kawamura, Hiroaki Tanaka, Ryota Tachibana, Kento Yoshikawa, Shintaro Maki, Kuniaki Toriyabe, Hiroki Takeuchi, Shinji Katsuragi, Kayo Tanaka, Tomoaki Ikeda

**Affiliations:** Department of Obstetrics and Gynecology, Mie University Graduate School of Medicine, 2-174 Edobashi, Tsu, Mie 514-8507 Japan

**Keywords:** Diseases, Health care

## Abstract

We aimed to investigate the effects of maternal tadalafil therapy on fetal programming of metabolic function in a mouse model of fetal growth restriction (FGR). Pregnant C57BL6 mice were divided into the control, L-NG-nitroarginine methyl ester (L-NAME), and tadalafil + L-NAME groups. Six weeks after birth, the male pups in each group were given a high-fat diet. A glucose tolerance test (GTT) was performed at 15 weeks and the pups were euthanized at 20 weeks. We then assessed the histological changes in the liver and adipose tissue, and the adipocytokine production. We found that the non-alcoholic fatty liver disease activity score was higher in the L-NAME group than in the control group (p < 0.05). Although the M1 macrophage numbers were significantly higher in the L-NAME/high-fat diet group (p < 0.001), maternal tadalafil administration prevented this change. Moreover, the epididymal adipocyte size was significantly larger in the L-NAME group than in the control group. This was also improved by maternal tadalafil administration (p < 0.05). Further, we found that resistin levels were significantly lower in the L-NAME group compared to the control group (p < 0.05). The combination of exposure to maternal L-NAME and a high-fat diet induced glucose impairment and non-alcoholic fatty liver disease. However, maternal tadalafil administration prevented these complications. Thus, deleterious fetal programming caused by FGR might be modified by in utero intervention with tadalafil.

## Introduction

Fetal growth restriction (FGR) is a condition in which the fetus does not reach its growth potential for a given gestational age. Studies have shown that the postnatal environment and lifestyle contribute to metabolic disease in adulthood; however, the prenatal environment is also important^1^. According to the fetal programming theory, metabolic diseases such as diabetes, hypertension, and dyslipidemia can be ascribed to the prenatal environment because injuries that occur during important developmental periods cause physiological and metabolic changes^2^. Thus, an inadequate intrauterine environment may lead to long-term adverse effects in adult life. Accordingly, infants with FGR are susceptible to type 2 diabetes mellitus and fatty liver disease in adulthood^3^. FGR is caused by a dysfunction in early placental development^4^, and subsequent postnatal rapid catch-up growth results in metabolic disease development.

L-NG-nitroarginine methyl ester (L-NAME) decreases the vasodilatory effect of nitric oxide (NO) and induces hypertensive disorders in pregnancy (HDP). Similar conditions can be produced in mice, and we previously reported that mice treated with L-NAME are suitable candidates for an FGR mouse model^4^. In that study, we speculated that tadalafil would indirectly promote fetal growth by causing dilatation of the maternal blood sinuses of the placenta^4^. The association between HDP and FGR has been recognized because it results from failed trophoblast invasion and uterine spiral artery remodeling, which leads to blood flow reduction, placental ischemia, and FGR^5^. Furthermore, NO produced by NO synthases regulates the placental vascular tone. Placental vessels express molecular mediators for the NO-dependent pathway, including cyclic guanosine monophosphate-specific phosphodiesterase 5 (PDE5)^6^. Recently, PDE5 inhibitors, which act by dilating arteries and increasing blood flow and are used to treat erectile dysfunction and pulmonary hypertension^6^, were suggested as a potential remedy for FGR^7^. Concurrently, our study showed that tadalafil, a selective PDE5 inhibitor, could be used in this manner^8–12^. However, the effect of L-NAME on the metabolic status of the offspring, as well as tadalafil’s potential to modulate such outcomes, has not been investigated. Therefore, in this study, we aimed to validate L-NAME-induced FGR in mice using a murine model that consisted of an inadequate intrauterine environment associated with metabolic disease. Additionally, we investigated the effects of maternal tadalafil administration for FGR on the prevention of high-fat diet-induced glucose intolerance and fatty liver disease in the offspring.

## Results

### Body and organ weights of the offspring

**Figure 1:**
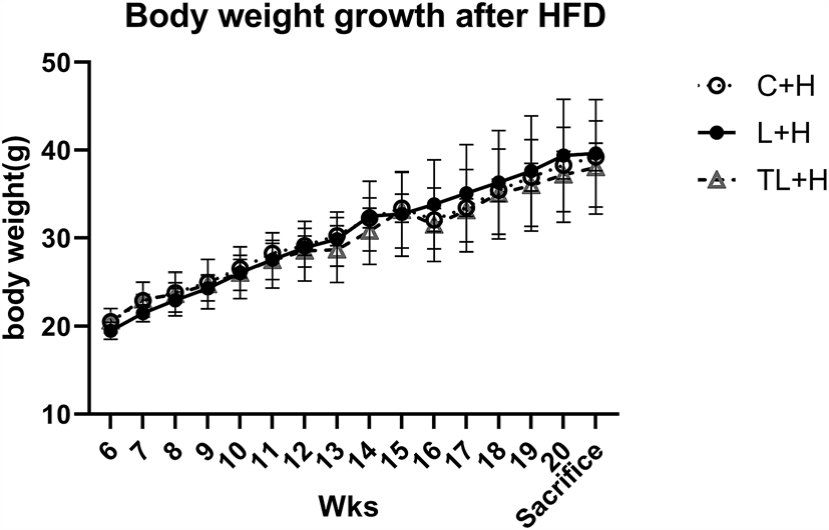
Postnatal body weights of the pups from different treatment groups. At the time of initial sampling, there were no significant differences in the bodyweights between the high-fat diet groups. The birth weight was not measured to reduce maternal stress. Data are presented as mean ± standard deviation, *n* = 4–7/group. A two-way repeated analysis of variance with a post hoc Tukey test was used for multiple comparisons. *C + H* control + high-fat diet, *HFD* high-fat diet, *L + H* L-NG-nitroarginine methyl ester + high-fat diet, *L-NAME* L-NG-nitroarginine methyl ester, *TL + H* L-NG-nitroarginine methyl ester + tadalafil + high-fat diet.

The offspring were divided into three groups based on the maternal intervention administered. These groups were as follows: control (C), treatment with L-NAME only (L), and treatment with both L-NAME and tadalafil (TL). The control group was not treated with L-NAME or tadalafil. In addition, since each offspring group received a high-fat diet (HFD) after 6 weeks, the term “ + H” was added after the three group names (C + H, n = 6; L + H, n = 4; and TL + H, n = 7). There were no significant differences in the weights of the mice who were given a HFD from 6 weeks until the day they were euthanized (Fig. 1). Further, the whole epididymal adipose tissue, liver, and pancreas weights per body weight (g/kg) did not differ significantly among the groups (Table 1). The blood pressures in the dams 16 days post-coitum (d.p.c.) were 102.3 ± 7.7 mmHg, 23.7 ± 10.7 mmHg, and 114.2 ± 6.8 mmHg in the C, L, and TL groups, respectively. A significant difference was noted between the C and L, and L and TL groups. The sex of the offspring in the C, L, and TL groups were 20, 14, and 23 male and 10, 21, and 8 female, respectively. The weights of the offspring within 48 h after birth were 1.56 ± 0.11 g, 1.38 ± 0.15 g, and 1.46 ± 0.11 g in the C, L, and TL groups, respectively (C–L, p = 0.0003; C–TL, p = 0.0322; and L–TL, p = 0.13). The weights of the offspring within 3 weeks of birth were 8.51 ± 0.68 g, 8.23 ± 0.59 g, and 8.04 ± 1.15 g in the C, L, and TL groups, respectively (C–L, p = 0.88; C–TL, p = 0.62; L–TL, p = 0.93). Within 5 weeks after the birth, the offspring weighed 19.26 ± 0.64 g, 18.82 ± 1.03 g, and 19.47 ± 1.21 g in the C, L, and TL groups, respectively (C–L p = 0.78, C–TL p = 0.92, L–TL p = 0.56).

**Table 1:** Analyses of tissues from mice of different treatment groups.

	C + H	L + H	TL + H
Body weight	39.22 ± 1.57	39.63 ± 6.11	38.02 ± 5.29
Liver	1.42 ± 0.11	1.39 ± 0.34	1.29 ± 0.37
Pancreas	0.19 ± 0.03	0.23 ± 0.04	0.19 ± 0.03
Femoral muscle	0.15 ± 0.02	0.14 ± 0.02	0.14 ± 0.02
Epididymal tissue	2.19 ± 0.25	2.82 ± 0.27	2.04 ± 0.46

*C + H* control + HFD, *L + H* L-NAME + HFD, *TL + H* L-NAME + tadalafil + HFD, *L-NAME* L-NG-nitroarginine methyl ester, *HFD* high-fat diet.

### GTTs

**Figure 2:**
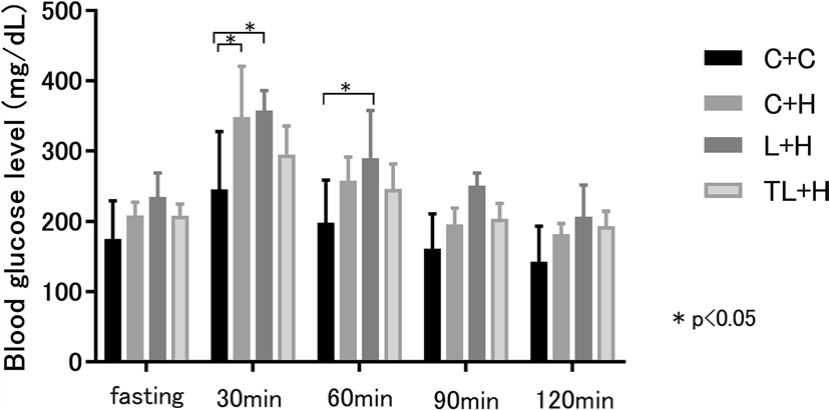
Glucose tolerance test results at 16 weeks in the treatment groups. At 15 weeks, glucose was administered intraperitoneally (1 mg/kg body weight) after an overnight fast. The blood glucose levels 30 min after glucose administration were significantly lower in the TL + H group than in the L + H group. Furthermore, the blood glucose levels at 90 min were higher in the L + H group than those in the other groups. Data are presented as the mean ± standard deviation, *n* = 4–7/group; **p < 0.01, *p < 0.05, based on a two-way repeated analysis of variance with a post hoc Tukey test for multiple comparisons. *C + H* control + high-fat diet, *HFD* high-fat diet, *L + H* L-NG-nitroarginine methyl ester + high-fat diet, *L-NAME* L-NG-nitroarginine methyl ester, *TL + H* L-NG-nitroarginine methyl ester + tadalafil + high-fat diet.

In terms of the fasting glucose levels, there were no significant differences noted between the groups (Fig. 2). However, the blood glucose levels 30 min after glucose administration were significantly lower in the TL + H group than in the L + H group (p < 0.05). Furthermore, blood glucose levels after 90 min were higher in the L + H group than those in the other groups (C + H, p < 0.01 and TL + H, p < 0.05).

### Adipocyte morphology

**Figure 3:**
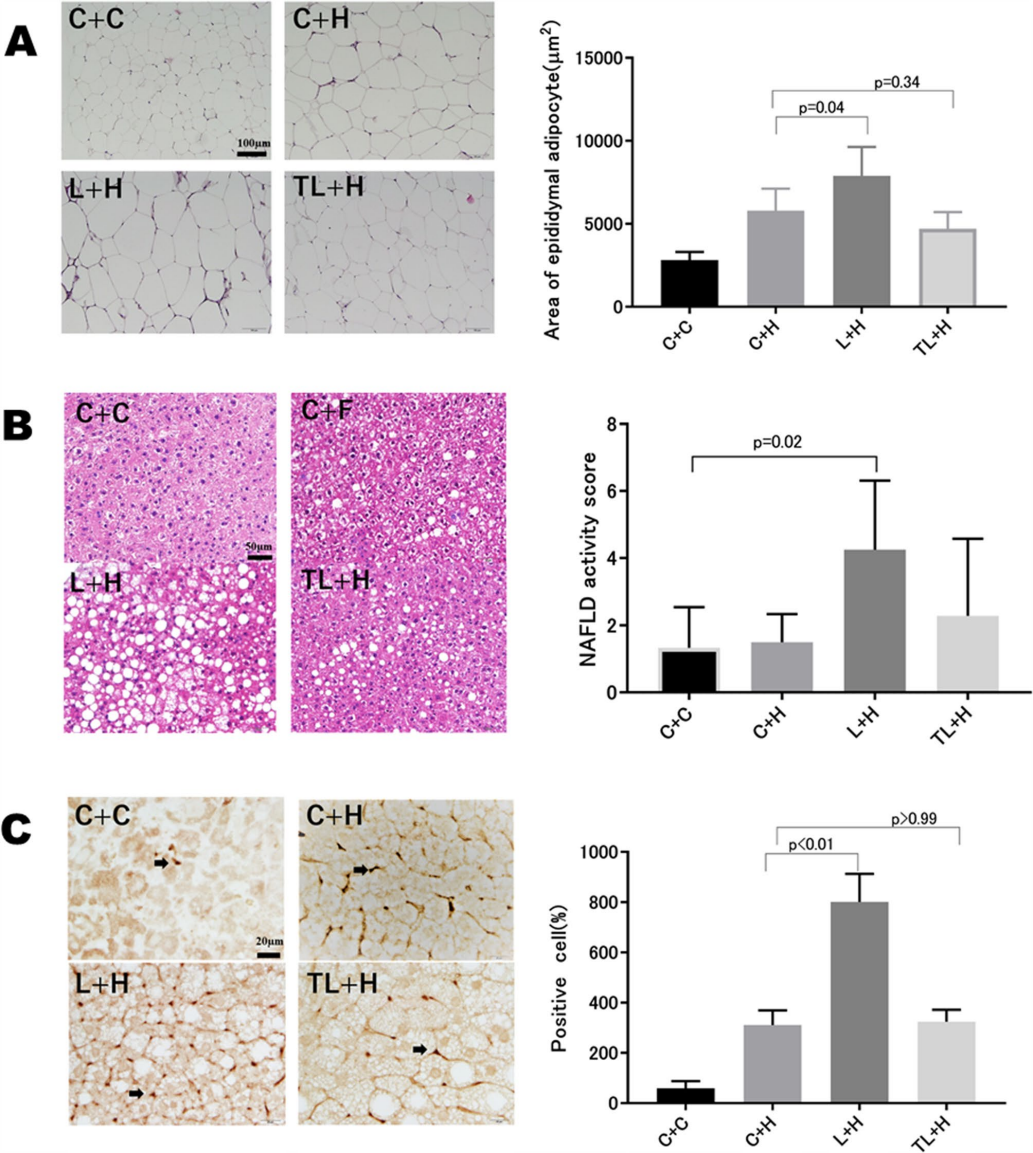
Histological examinations of tissues from the different treatment groups. (**A**) A liver at 20 weeks postnatally (HE stain, × 200 magnification). (**B**) Epididymal fatty cell area (HE stain, × 200 magnification). (**C**) F4/80 immunohistochemistry (arrow: positive cells, × 400 magnification). Morphologic changes in the liver and adipose tissue due to tadalafil and L-NAME administration. C + C indicates treatment naïve mice to whom no maternal intervention or HFD was given. Data are presented as the mean ± standard deviation, *n* = 4–7/group. ***p < 0.001, **p < 0.01, *p < 0.05, based on a one way-analysis of variance with a post hoc Tukey test for multiple comparisons. *C + C* control + control diet, *C + H* control + HFD, *HE* hematoxylin and eosin, *HFD* high-fat diet, *L + H* L-NG-nitroarginine methyl ester + high-fat diet, *L-NAME* L-NG-nitroarginine methyl ester, *NAFLD* non-alcoholic fatty liver disease, *TL + H* L-NAME + tadalafil + HFD, *TL + H* L-NG-nitroarginine methyl ester + tadalafil + high-fat diet.

The mean epididymal adipocyte size was significantly larger in the L + H group than in the C + H and TL + H groups (p < 0.05 and p < 0.01, respectively; Fig. 3A).

### Morphological changes in the livers of the offspring

The mean non-alcoholic fatty liver disease (NAFLD) activity score (NAS) was higher in the L + H group than in the C + H group (p < 0.05), whereas there were no significant differences between the C + H and TL + H groups (Fig. 3B). Additionally, the F4/80-positive cell count was higher in the L + H group than in those in the other groups (p < 0.001; Fig. 3C).

### Enzyme-linked immunosorbent assay (ELISA)

**Figure 4:**
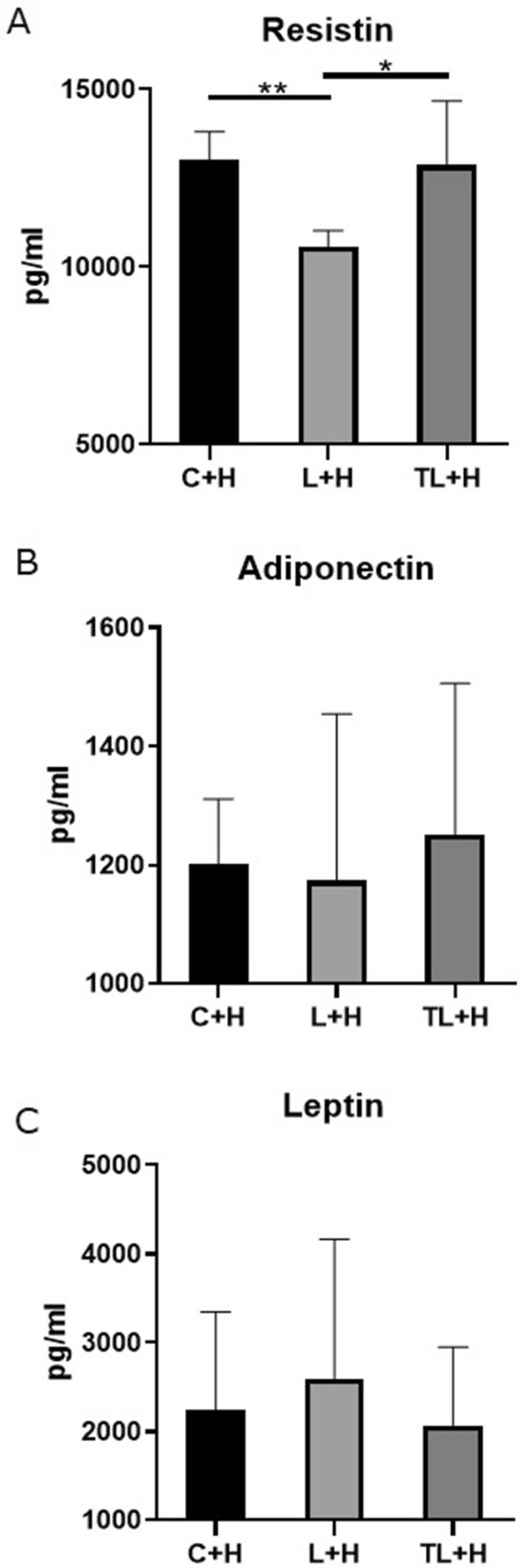
Results of the ELISA for adipocytokines among the different treatment groups. (**A**) Plasma resistin, (**B**) plasma adiponectin, and (**C**) plasma leptin. Data are presented as mean ± standard deviation, *n* = 4–7/group; **p < 0.01, *p < 0.05 based on a Welch analysis of variance with a post-hoc Games-Howell test for multiple comparisons. (**C**) *C + H* control + high-fat diet, *HFD* high-fat diet, *L + H* L-NG-nitroarginine methyl ester + high-fat diet, *L-NAME* L-NG-nitroarginine methyl ester, *TL + H* L-NG-nitroarginine methyl ester + tadalafil + high-fat diet, *ELISA* enzyme-linked immunosorbent assay*.*

The serum resistin levels in the L + H group were lower than those in the C + H and TL + H groups (p < 0.05, Fig. 4). However, the serum adiponectin and serum leptin levels did not differ significantly between the groups (Fig. 4).

## Discussion/comment

### Principal findings

Our study found that maternal L-NAME administration affected both glucose tolerance, and liver and adipose tissue morphology in adult mice offspring. Furthermore, these changes could be prevented through the administration of maternal tadalafil therapy.

### Results

In this study, fatty liver disease was observed in the offspring of mice with L-NAME-induced FGR. However, the maternal tadalafil therapy results suggested that in utero interventions can be used to modify fetal programming. In addition, we found that these changes could cause glucose intolerance in the L-NAME group but this outcome was partially prevented by tadalafil treatment. Furthermore, mice with L-NAME-induced FGR can be used as a model for preeclampsia as they result in abnormal metabolism in adulthood. An insulin-resistant preeclampsia model (performed at 40 weeks) that used angiotensin 2 type 1 receptor antibodies and a high-sugar diet at 40–48 weeks found that fasting insulin levels were also high. In this model, the researchers observed a remarkable increase in the fasting blood glucose levels compared to the controls^13^. Moreover, offspring with FGR undergo catch-up growth and develop insulin resistance with elevated insulin-like growth factor 1 levels during adulthood as a result of these changes^14^. Our results suggest that maternal tadalafil administration can potentially prevent FGR-related glucose intolerance.

NAFLD occurs as a result of dysregulated lipid homeostasis, which is primarily attributed to an increase in hepatic fatty acid lipogenesis, and a decrease in fatty acid β-oxidation and lipid output^15^. A previous report showed that the lipid content and messenger ribonucleic acid (mRNA) expression of lipogenesis-associated genes are enhanced in growth-restricted fetuses and neonates^16^. These results suggest that FGR might lead to dyslipidemia and obesity in adulthood^17^. Furthermore, the prevalence of type 2 diabetes is increased in obese and non-obese patients who have been diagnosed with NAFLD^18^. Other features of NAFLD include hyperglycemia 2-h after glucose loading, even when the fasting blood glucose levels are normal, and hyperinsulinemia with decreased hepatic insulin clearance (confirmed with a large patient cohort). Furthermore, glucose intolerance is correlated with the NAS^19^. In this study, the combination of in utero FGR and postnatal administration of an HFD induced histological changes in the livers of the offspring, whereas tadalafil suppressed these changes. Moreover, we showed that the mean NAS in the L + H group alone exceeded 4 points, which indicated severe NAFLD^20^.

In children with NAFLD, numerous activated macrophages are located in the spaces between the damaged hepatocytes. Moreover, macrophages can be used to predict progressive NAFLD before the onset of inflammation or fibrosis^21^. Here, we observed an increase in the F4/80-positive cell count, an M1 macrophage marker, in the L + H group. However, maternal tadalafil ameliorated this change.

Otherwise, adipocytokines are one of the most important contributing factors to glucose intolerance^22^. We used ELISA to show that L-NAME could induce adipocytokine changes. Although an increase in the serum resistin levels can induce insulin resistance^23^, L-NAME administration decreased serum resistin levels. Previous research has reported that L-NAME induces resistin mRNA expression and improves insulin sensitivity in adults, which may be the reason for this result^24^. In this case, maternal L-NAME administration may change resistin mRNA expression in both the mothers and the pups. Moreover, these levels returned to control levels following maternal tadalafil administration. Despite these results, the glucose tolerance in the offspring improved. In a previous study, obese mice showed a decrease in their serum resistin levels; however, these results were controversial^25^. Thus, further investigation on the relationship between resistin and glucose tolerance in obese individuals is needed.

In this study, the area of the adipocytes in the epididymal tissue was lower in the maternal tadalafil group than in the L-NAME group. In addition, L-NAME administration aggravated adipocyte enlargement. Previous studies found that giant adipocytes in in vivo visceral adipose tissue were resistant to the antilipolytic effects of insulin^26^. Increased adipocyte size may be caused by decreased adiponectin secretion, increased free fatty acid release, and imbalanced proinflammatory and inflammatory cytokine production. This is called adipose tissue dysfunction and it can be deleterious in terms of changes in insulin sensitivity^27–29^. In addition to regulating systemic energy storage, adipose tissue secretes a number of adipokines that significantly affect lipid homeostasis and insulin resistance^30^. The decrease in the adipocyte size in the maternal tadalafil group suggests that this drug could be used to improve insulin resistance.

### Strengths and limitations

Our study found that maternal tadalafil can alter the metabolism of adult mice offspring. This pharmacological agent modulated the liver and adipose tissue morphology and improved glucose intolerance. Previously, we focused on the effects of maternal tadalafil on birth weight and neurological defects, but we considered the need to address its effects on the metabolism of adult mice offspring. However, this study has several limitations. First, we did not investigate gene expression. We propose that maternal tadalafil therapy alters gene expression in the pre- and postnatal periods. However, such alterations and the mechanism associated with the effects of tadalafil have not been studied. These topics should be addressed in future studies. Second, we did not provide adequate investigation into the effects on the pancreas and, in our next study, we plan to address pancreatic function. Third, there has been no investigation into cases without a second hit. It is crucial that we develop an understanding of this point as tadalafil can potentially induce abnormal gene expressions that might have unfavorable effects. Hence, the clinical applicability of this study for humans should be considered with caution.

Alterations to the metabolic profiles caused by a combination of FGR and HFD were prevented through maternal tadalafil treatment, which suggests that in utero intervention could modify fetal metabolic programming to yield favorable results.

### Clinical implications

Although the mortality rate for fetuses with FGR has improved, this condition complicates fetal development and future adult diseases, resulting in significant problems. Therefore, the prevention of such issues by intervening during the fetal period is of great importance. In this study, our results suggest that maternal tadalafil therapy could minimize the effects of HFD as a postnatal second hit by preventing alterations in fetal programming, which is the inherent first hit.

## Methods

### Nonhuman experimentation

The experimental protocol was approved by the Ethics Committee for Animal Research of the Mie University Graduate School of Medicine (Institutional Review Board number; 29-20). All methods were carried out in accordance with the relevant guidelines and regulations.

### Animals

**Figure 5:**
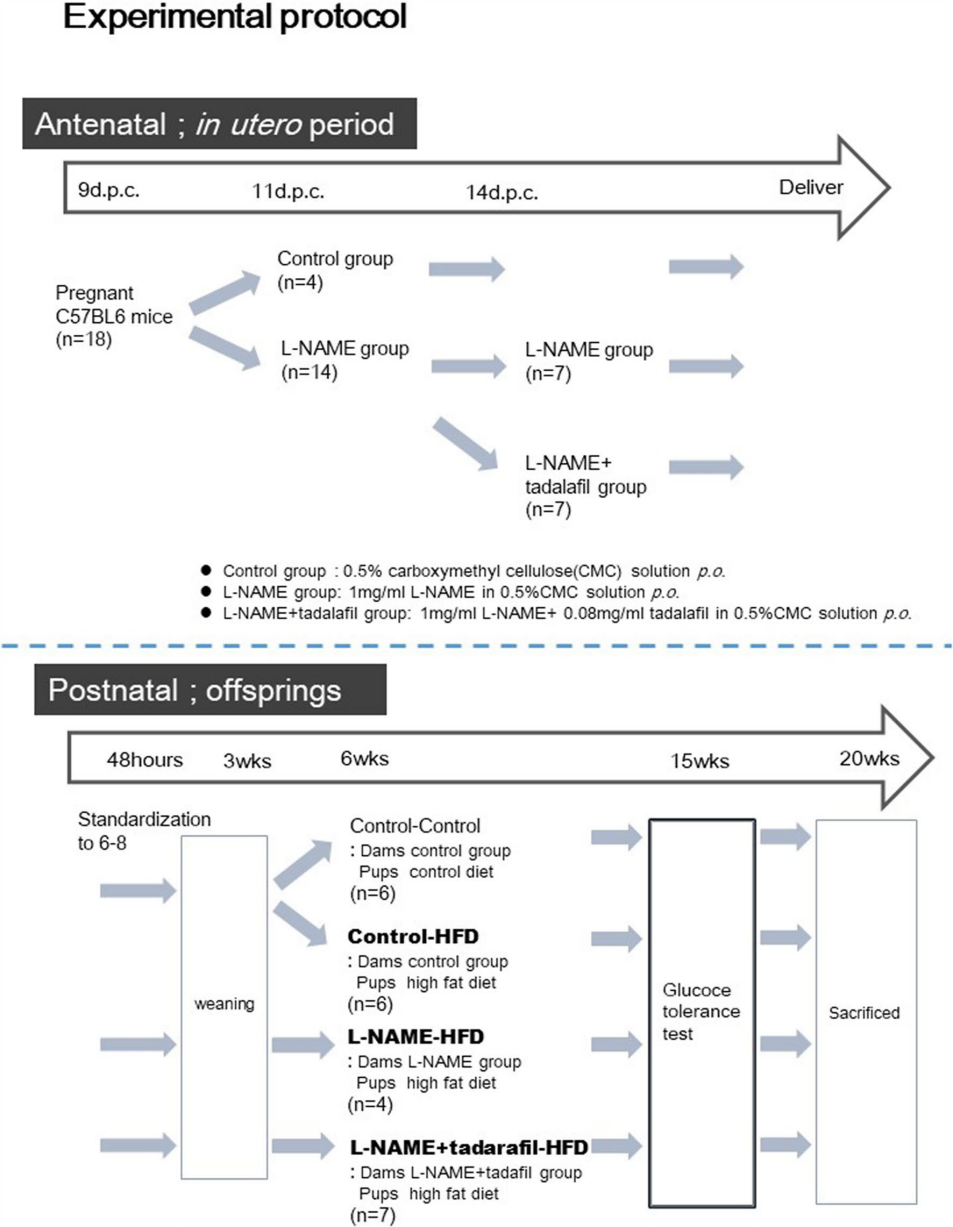
Experimental protocol. The litters were standardized to 6–8 pups per litter 48-h post-partum, allowing the pups to receive equal amounts of milk and maternal pup care. All dams were given normal drinking water during lactation. After 6 weeks, the male pups from each group were fed an HFD and treated as follows: control + HFD (C + H, n = 6), L-NAME + HFD (L + H, n = 4), and L-NAME + tadalafil + HFD (TL + H, n = 7). *HFD* high-fat diet, *L-NAME* L-NG-nitroarginine methyl ester.

The experimental protocol is shown in Fig. 5. We used the same protocol as the one that we previously described for a mouse model for L-NAME-induced FGR using a C57BL6 background^12^. C57BL6 mice (CLEA, Tokyo, Japan) were purchased 9 d.p.c. The animals were housed individually in a temperature- and humidity-controlled facility with automatically controlled 12-h light and dark cycles. The mice were provided with food and water ad libitum, and the intake and body weight of the pregnant mice were evaluated daily.

The pregnant mice were allocated to the control or L-NAME groups at 11 d.p.c. The groups were matched by weight. The control group received 0.5% carboxymethylcellulose (CMC; Wako Pure Chemical Industries, Osaka, Japan) dissolved into their drinking water. The L-NAME group received 1 mg/ml of L-NAME (Cayman Chemical Company, Ann Arbor, MI, USA) dissolved into 0.5% CMC. The dams that were treated with L-NAME were allocated to the two subgroups at 14 d.p.c. The subgroups were matched by their weight and systolic blood pressure (SBP). SBP was measured using a tail-cuff microsensor connected to a non-invasive blood pressure monitor for mice (MK-2000A; Muromachi Kikai, Tokyo, Japan).

One subgroup continued to only receive L-NAME, whereas the other subgroup received L-NAME with 0.08 mg/ml of tadalafil (Cayman Chemical Company, Ann Arbor, MI, USA) suspended in 0.5% CMC. Maternal SBP measurement was repeated at 16 d.p.c.

The litters were standardized to six to eight pups per litter after 48-h post-partum to ensure that the animals received equal quantities of milk and maternal pup care. To minimize human interference and prevent the hinderance of lactation and care, the birth weights were not measured. All dams were provided with normal drinking water during lactation. After 6 weeks, the male pups from each group were chosen randomly to receive an HFD (4.73 kcal/g, with fat comprising 45% of the total calories, and consisting of soybean oil [5.6%], lard [39.4%], and a 20% protein formula; D12451, Research Diets, New Brunswick, NJ, USA). Thus, the three groups of offspring based on the maternal interventions were as follows: control + HFD, L-NAME + HFD, and L-NAME + tadalafil + HFD (C + H, n = 6; L + H, n = 4; and TL + H, n = 7, respectively). Only the male pups were used for the experiments because, with this model, HFD-related metabolic disorders are more prevalent in males than in females^31^. The weights of the pups were recorded and the animals underwent GTTs at 15 weeks. They were euthanized at 20 weeks, after which blood collection and tissue sampling were performed.

### Tissue sampling

The whole liver, pancreas, right femoral muscle, and epididymal tissues were dissected and weighed. Standard procedures were used to fix some liver and epididymal adipose tissue in 4% paraformaldehyde (Nacalai Tesque, Inc.; Kyoto; Japan) in a 0.2 M sodium phosphate buffer (PBS; pH 7.4) and embedded in paraffin (Merck Ltd., Frankfurter, Germany).

### Glucose tolerance testing

At 15 weeks, after the mice were fasted overnight, a GTT was performed through intraperitoneal glucose administration (1 mg/kg body weight). The GTTs were performed during the light phase. Blood samples were taken from the tip of the tail, and the blood glucose concentration was determined using a OneTouch Ultra (Johnson & Johnson, Tokyo, Japan)^32^.

### Adipocyte size

A section of epididymal adipose tissue was fixed in 4% formalin in PBS, embedded in paraffin, and stained with hematoxylin and eosin. The fields of vision were selected randomly. The diameters of each adipocyte in each field were measured manually, and the diameters of 100 adipocytes were measured microscopically by a single observer^31,33^.

### NAS

A section of liver tissue was fixed in 4% formalin in PBS, embedded in paraffin, and stained with hematoxylin and eosin. The fields of vision were selected at random and scored based on the NAS, which consisted of steatohepatitis, lobular inflammation, and hepatocyte ballooning^20^.

### Immunohistochemistry

As previously described, a section of liver tissue was fixed in 4% formalin in PBS and embedded in paraffin using an avidin–biotin-peroxidase complex technique and fluorescent immunostaining after microwave antigen retrieval^34^.

The sections were incubated at room temperature overnight with an anti-F4/80 antibody (1:250, Santa Cruz Biotechnology, Dallas, TX, USA) and successively treated with a biotinylated secondary antibody and peroxidase-avidin complex (ABC-kit; Vector Laboratories, Burlingame, CA, USA). Five images of the specimen were randomly separated and digitally captured at 400× magnification. The number of positive cells was then counted and assessed as the number per high-power field^35,36^.

### ELISA

Serum adiponectin and resistin were determined using the Mouse Serum Adiponectin Quantikine ELISA (R&D systems, Minneapolis, Min, USA). Serum leptin was analyzed using the Mouse Serum Leptin kit (Shibayagi, Tokyo, Japan).

### Statistical analysis

All values are presented as mean ± standard deviation. All statistical analyses were conducted using GraphPad Prism8 (Graphpad, San Diego, CA, USA). Data were tested for equality of variances and then analyzed either by one-way analysis of variance (ANOVA), Welch ANOVA with a post hoc Tukey test for multiple comparisons, or with a Games-Howells test. We considered p-values < 0.05 as statistically significant. GTTs and bodyweights were analyzed by two-way repeated ANOVA with a post hoc Tukey test for multiple comparisons.
